# Optimizing Steam Consumption of Mushroom Canning Process by Selecting Higher Temperatures and Shorter Time of Retorting

**DOI:** 10.1155/2020/6097343

**Published:** 2020-03-28

**Authors:** Didik J. Pursito, Eko H. Purnomo, Dedi Fardiaz, Purwiyatno Hariyadi

**Affiliations:** ^1^Department of Food Science and Technology, Faculty of Agricultural Engineering and Technology, IPB University (Bogor Agricultural University), 16680, Indonesia; ^2^Indonesian Food and Drug Authority (Indonesian FDA), 10560, Indonesia; ^3^Southeast Asian Food and Agricultural Science and Technology (SEAFAST) Center, IPB University (Bogor Agricultural University), 16680, Indonesia

## Abstract

Increasing energy cost has driven the food canning industries to optimize their energy consumption in order to produce safe and shelf-stable foods efficiently. In the mushroom canning industry, energy efficiency is very critical to improve product (price) competitiveness. This research aimed at demonstrating total steam consumption to achieve the same sterility level (*F*_0_-value) of canned mushroom by using different combinations of times and temperatures of retorting. *Agaricus bisporus* in brine contained in 300 × 407 cans was heat processed in a horizontal static retort. Three different retort temperatures (115, 121, and 130°C) and different operator processing times ranging from 2 to 97 minutes were employed to achieve different levels of *F*_0_-values. Our results showed that at the same level of sterility, steam consumption inversely decreased with the increase of retort temperature. At the same *F*_0_-value of 10 minutes, energy efficiency for up to 72.9% and 58.1% per batch of retorting was achieved by increasing the temperature from 115 to 130°C and 115 to 121°C, respectively. Since steam consumption is a major element of production costs in the canning industry, the selection of higher temperatures and shorter time of retorting will have a positive commercial impact due to the reduction of production costs.

## 1. Introduction

Thermal processing is one of the most cost-effective food processing and preserving techniques and widely used in the food industry [1, 2]. It is an important technique not only to preserve food products by extending the shelf life, but also to improve eating quality and increase food availability, security, and affordability [3]. Food products are heated for a certain period and temperature to achieve the safety standard of the commercial sterilization condition [4], where retorting is the most common approach [5].

Steam consumption in sterilization process using retort is a major element of production cost in the canned food manufacturing [6]. A crucial issue in the food canning industry is an effort to minimize production costs by reduction of steam consumption in the sterilization process [7, 8]. The facts show that food canning industries that carry out sterilization processes with batch retorts tend to overcook it in ensuring the achievement of sterile commercial conditions. [9–12]. Excessive heating must be avoided due to decreased product quality and underutilized plant capacity [13–15]. Unnecessary overprocessing may lead to steam overconsumption that can significantly increase production costs [16]. Therefore, thermal processing should be optimized in order to minimize energy consumption and deterioration of desirable quality characteristics and to achieve the desired shelf-life of food products due to the elimination of spoilage microorganisms [17, 18].

Various thermal process literatures are available but mostly related to the point of view of microbiological and biochemical aspects. Studies on energy management to optimize the steam consumption conducted by Bhowmik and Hayakawa [19], Simpson et al. [20], Kannan and Neeharika [21], and Holdsworth and Simpson [22] provided examples of theoretical evaluations and experimental models, as well as energy consumption practices on a laboratory scale of thermal processing. However, the study of steam consumption in the retorting process at various temperatures and *F*_0_-values in the real food industry system is still limited.

We selected mushroom as a model of study because mushroom *(Agaricus bisporus)* is one of the popular canned food products in the global market [23] and recognized for its nutraceutical properties [24]. However, *Agaricus bisporus* is highly perishable and rapidly lose their sensory characteristics [25], also extremely sensitive to heating. This research aimed at optimizing total steam consumption to achieve the same *F*_0_-value of canned mushroom by using the different combinations of times and temperatures of retorting. We hope this research will increase the competitiveness of mushroom canning industries by improvement in optimizing design and operation with more efficient steam consumption.

## 2. Materials and Methods

### 2.1. Materials

The raw material was fresh *Agaricus bisporus* mushrooms, which were cultivated by PT. Suryajaya Abadiperkasa, Probolinggo, East Java, Indonesia. Devices used were a horizontal static retort with a diameter of 1.25 m, length 2.35 m (Chi Yinfa, Taiwan), intelligent digital vortex flow meter, and flow totalizer (Yantai Auto Instrument Making Co. Ltd). We used 11 pieces of temperature data logger OM-CP-Hitemp140 and two high-temperature data loggers with a flexible probe and OM-CP-Hitemp140-PT (Omega Engineering, Norwalk, Connecticut, USA). Both OM-CP-Hitemp140 and OM-CP-Hitemp140-PT data loggers are high-precision device, submersible, and can withstand temperatures up to 140°C (284°F), equipped with probes with the accuracy of ±0.1°C (0.18°F) over the entire operating range of -200 to 260°C (-328 to 500°F). Steam was produced by a Boiler (Omnical GmbH No. 16496, Germany) with capacity of 3,000 kg/h, maximum temperature of 160°C, and maximum pressure of 6 bar.

### 2.2. Methods

Preliminary experiments carried out to find the coldest point in the retort crates and sterilizers in view of observing various positions utilizing data loggers (heat distribution test). This study was carried out by evaluating the adequacy of the heating process in canned products from *Agaricus bisporus*. Adequacy of the sterilization process was assessed based on heat penetration data and calculated as a minimum *F*_0_-value. The procedure for implementing the heat distribution test referred to the Institute for Thermal Processing Specialists (IFTPS) protocol [26]. A total of eleven wireless data loggers were distributed in a retort at the center of the can to measure heat penetration.

The retort consists of three baskets where cans are loaded jumbly to the basket. Each basket was filled with 700 cans of whole mushroom products or in full capacity conditions. Before the experiment was carried out, the condensation fluid in the retort was removed. Retort operators maintained other sterilization parameters (e.g., temperature and pressure) and carried out steps as instructed in the canning factory standard operating procedures. For heat penetration tests, all products tested were prepared using the standard operating procedures practiced at the respective food canning establishments. All products tested had solid elements of mushrooms as the major components so that it was assumed that packs tested were heated mainly by conduction. Data loggers were located at the center of the cans and their tips placed inside the rigid part of the mushroom product. The *Agaricus bisporus* products tested with attached data loggers were then strategically located near the center of each basket. Steam consumption was measured using a vortex flowmeter [27].

### 2.3. Canning Process Condition

**Figure 1:**
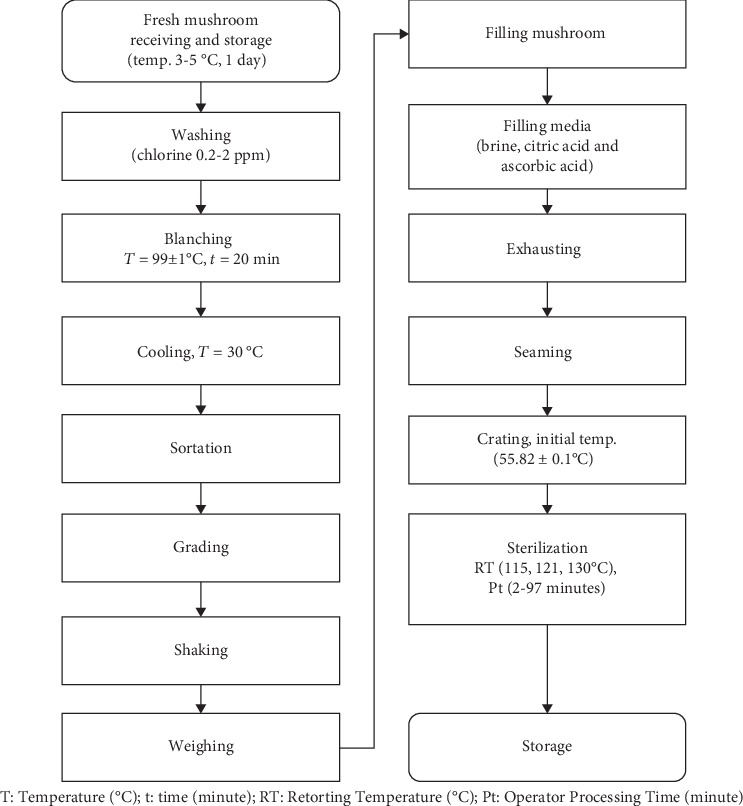
Process flow diagram for canned *Agaricus bisporus.*

The canning process carried out in this study was using standard processing steps in the mushroom canning industry, as in Figure 1. In general, the steps of the mushroom canning process were the preparation of materials, blanching, filling into cans, filling medium, exhausting, seaming, sterilization, cooling, labeling, and storage. This experiment produced canned products from brined *Agaricus bisporus* in sizes of 8 oz cans with dimensions of 300 × 407.

### 2.4. *F*_0_-Value Calculations

Retort temperatures used for sterilization were 115, 121, and 130°C. In this study, the average initial temperature of the product used was 55.82 ± 0.1°C, and the come-up time retort was 12 minutes. Heat penetration test was conducted using the calibrated Omega wireless data loggers, USA. Sterility was expressed as an *F*_0_-value calculated using General Method [14], as in

(1) $$\begin{array}{c} Fo=\int_{0}^{t}10^{T-T_{\text{ref}}/z}\text{dt}, \end{array}$$

where *F*_0_ is the heat adequacy for the commercial sterilization process, which is expressed as the equivalent heating time (in minutes) at a constant temperature of 121.1°C (250°F) to inactivate *Clostridium botulinum* spores. *T* is the temperature at any given time; *T*_ref_ is a reference processing temperature (121.1°C or 250°F), and *z*-value is 10°C.

**Figure 2:**
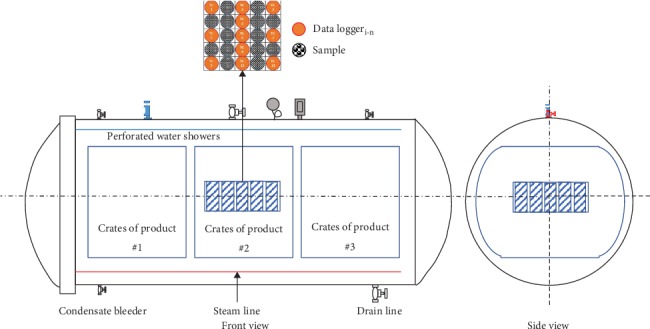
Schematic of data loggers in the retort.

Data loggers were installed in the predetermined location in the retort, as illustrated in Figure 2. The cooling area was discovered in the middle of the retort tray and the center of retort. The coldest point of its contents was assumed to be the geometric axis of the mushroom can. A total of eleven data loggers were used to measure heat penetration.

### 2.5. Measurement of Steam Consumption

**Figure 3:**
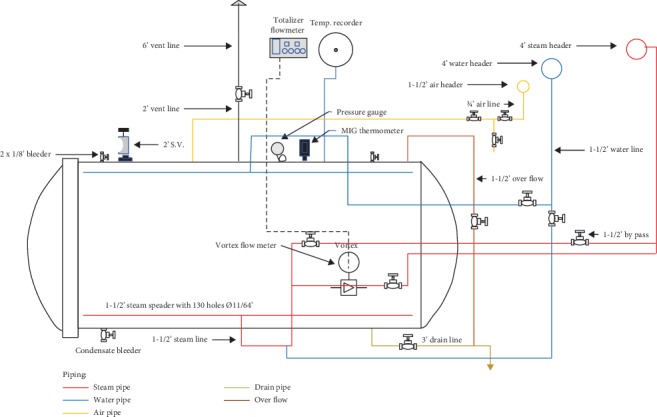
Schematic of retort system with the installation of the vortex flow meter and totalizer flow meter.

Steam consumption was measured by the vortex flowmeter, which was integrated with digital totalizer. The total consumption of steam is measured from “steam-on” to “steam-off” or during the come-up time and holding period. The cooling process was not measured because no steam was needed [20]. Steam consumption for each procedure was plotted against *F*_0_-values and retort temperatures to describe the relationship between those parameters. Vortex flowmeter mounted on inlet steam, as in Figure 3.

## 3. Results and Discussion

Shelf-stable canned *Agaricus bisporus* is categorized as commercial sterile foods because of having pH ≥ 4.6, a_w_ ≥ 0.85, packed hermetically, and thermally processed. A commercial sterility condition can be achieved by heat and other treatments sufficient to inactivate of spores to make the food free from microbes that can grow at room temperature (nonrefrigerated) during distribution and storage. Commercial sterility is generally set at a minimum *F*_0_-value of 12D values to provide a technical 12 log cycle reduction of the viable spores of the most heat-resistant microbe [28]. The condition of the commercial sterilization process is very dependent on various factors, such as pH, initial microbial load, species of microorganism, heat transfer in processed products and containers, media, and storage conditions after sterilization.

**Figure 4:**
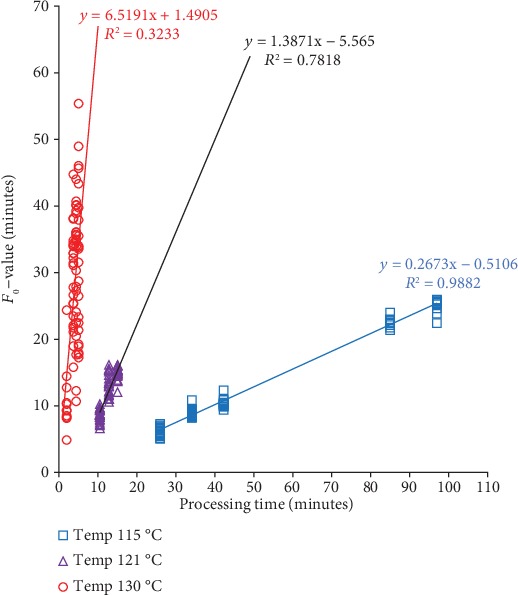
Effect the operator processing time on *F*_0_-value from different retort temperatures (115,121, and 130°C).

**Figure 5:**
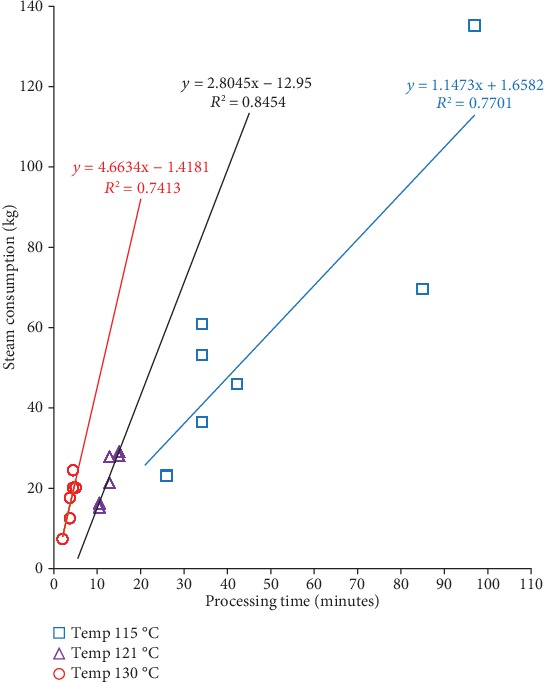
Effect the operator processing time on the steam consumption from different retort temperatures (115,121, and 130°C).

**Figure 6:**
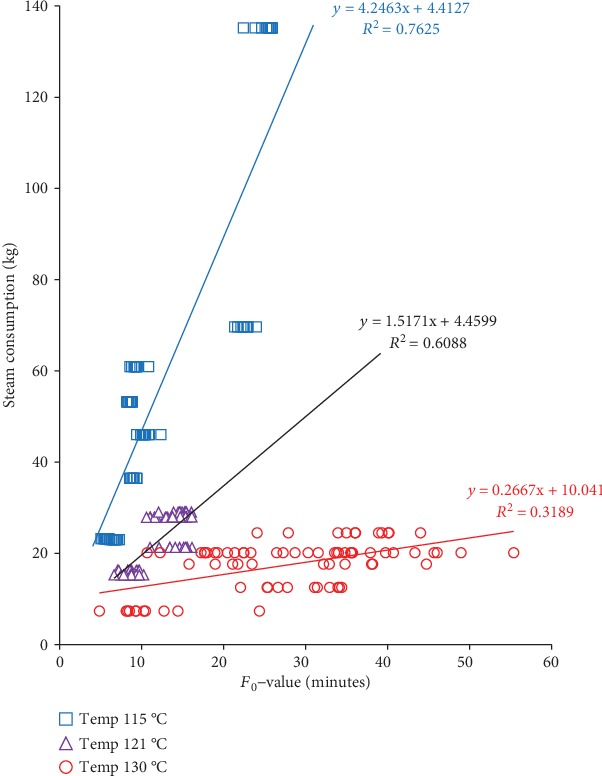
Effect *F*_0_-value on steam consumption from different retort temperatures (115,121, and 130°C).

We examined the relationship between steam consumption, *F*_0_-values, and operator processing time of sterilization at three different retort temperatures, as in Figures 4–6. The *F*_0_-value becomes the primary consideration in determining the combination of temperature and heating time in the sterilization process. The same *F*_0_-value can be obtained from different combinations of temperatures and sterilization times. Increasing temperature significantly reduces the time needed to achieve the same level of *F*_0_-value (see Figure 4). The estimated sterilization time required to achieve the desired *F*_0_-value can be calculated using obtained regression in each temperature. For the whole mushroom in the brine of *F*_0_-value of 10 minutes, the estimated sterilization time for temperature 115, 121, and 130°C was 39.32, 11.22, and 1.30 minutes to achieve the recommended *F*_0_-value, respectively.

The rate of *F*_0_-values can be determined from the gradient of each regression line (see Figure 4). The rate of *F*_0_-values increase is faster at a higher retort temperature. The highest rate of *F*_0_-values at a retort temperature of 130°C follows the General Method formula (see Equation (1)). The *F*_0_-value is a function of the time and process temperature. The *F*_0_-values depend more on process temperature than on process time [2, 29].

An increase in processing time caused an increase in steam consumption at all retort temperature conditions (115, 121, and 130°C), as in Figure 5. The rate of steam consumption can be determined by the gradient of the slope line, which describes the amount of steam consumption per minute of processing time. Based on the regression obtained, as in Figure 5, the steam consumption rate for 115, 121, and 130°C were 1.15, 2.80, and 4.66 kg/min, respectively. It is clear that in the same processing time, the steam consumption rate is higher with the higher retort temperatures.

However, operating retort with higher temperature results in less time required to obtain the same level of *F*_0_-value. Thus, the steam consumption needed may be determined by the combination of the steam consumption rate and operation required. For example, achieving the recommended *F*_0_-value of 10 minutes at 115, 121, and 130°C would take the operating time of 39.32, 11.22, and 1.30 minutes, therefore, require steam of more or less 45.11, 31.47, and 6.08 kg, respectively. It indicates that less steam is needed to obtain the same *F*_0_-value at a higher temperature.

The higher *F*_0_-value requires higher steam consumption at all retort temperatures. To achieve the same *F*_0_-value, the use of a higher retort temperature (130°C) requires the least steam consumption, as in Figure 6. This phenomenon occurred because the processing time of operators between three retort temperatures (115, 121, and 130°C) to achieve the same *F*_0_-value was different. At lower retort temperatures, the amount of steam consumption was higher because of the prolonged holding period. After reaching the retort temperature target or holding period, the bleeder and drain valve remain fully opened, while the steam was injected into the retort until the pressure was equivalent to the vapor pressure at the retort to maintain the retort temperature set. The sterilization process with a lower temperature will require a longer operator processing time to reach the same *F*_0_-value. The longer processing time will cause more significant heat loss. During a holding period, steam is used more to compensate for the amount of heat loss due to the bleeder, condensate, convection, and radiation [30].

A similar indication was reported by Bhowmik and Hayakawa [19] when they conducted a research using food simulant at two different retort temperatures (110°C and 121°C). They reported that at the same *F*_0_-value, steam consumption at 121°C was less than that of 110°C. Our research results provide more clarity since we used a wider range of retorting temperatures (at three different retort temperatures of 115, 121, and 130°C) and steam consumption data we measured in the real system of the canning industry.

## 4. Conclusions

Based on steam consumption, we found that the use of a higher temperature and shorter time is more economical compared to that of lower temperature and longer time of retorting. For canning process requiring the *F*_0_-value of 10 minutes, we found that the steam consumption efficiency for up to 72.9% and 58.1% per batch of retorting were achieved by increasing the temperature from 115 to 130°C and 115 to 121°C, respectively.

## Data Availability

Data will be provided upon submitted to the corresponding author discussion.
